# Deep Learning Enabled Optimization and Mass Transfer Mechanism in Ultrasound-Assisted Enzymatic Extraction of Polyphenols from Tartary Buckwheat Hulls

**DOI:** 10.3390/foods14162915

**Published:** 2025-08-21

**Authors:** Yilin Shi, Yanrong Ma, Rong Li, Ruiyu Zhang, Zizhen Song, Yao Lu, Zhigang Chen, Yufu Wang, Yue Wu

**Affiliations:** 1 College of Food Science and Technology, Nanjing Agricultural University, Nanjing 210095, China; s16638561632@163.com (Y.S.); myr18734409534@163.com (Y.M.); 9231810313@stu.njau.edu.cn (R.L.); 9231810318@stu.njau.edu.cn (R.Z.); 9231810315@stu.njau.edu.cn (Z.S.); 9231810319@stu.njau.edu.cn (Y.L.); wu.yue@njau.edu.cn (Y.W.); 2 School of Chemistry, The University of Melbourne, Parkville, VIC 3010, Australia

**Keywords:** ultrasound-assisted enzymatic extraction, Tartary buckwheat hulls, polyphenols, deep learning, numerical simulation

## Abstract

Tartary buckwheat hulls, a phenolic-rich by-product of buckwheat processing, offer great potential for resource utilization. In this study, ultrasound-assisted enzymatic extraction with two temperatures (40 °C and 50 °C) was employed to obtain phenolics from Tartary buckwheat hulls. Compared with the traditional extraction method (207 mg/100 g), ultrasound-assisted enzymatic extraction increased the total phenolic yield by 91.3% at 50 °C. Numerical simulations based on Fick’s law indicated that enzyme pretreatment concentration positively correlated with the effective diffusion coefficient ($D_{e}$), which increased from 9.15 × 10^−7^ to 2.00 × 10^−6^ m^2^/s at 40 °C. Meanwhile, the neuro-fuzzy inference system (ANFIS) successfully predicted the extraction yield under various ultrasonic conditions (R^2^ > 0.98). Regarding quantitative analysis of phenolic compounds in extracts, the results revealed that catechins and epicatechins were the most abundant in Tartary buckwheat hull. Additionally, phenolic acids rapidly diffused at higher temperatures (50 °C), and flavonoids were highly sensitive to temperature and enzyme synergy. Phenolic extracts exhibit significant potential for value-added applications in food processing, particularly in improving antioxidative stability, prolonging shelf life. This study provides a theoretical basis for green, efficient phenolic extraction from plant residues.

## 1. Introduction

Tartary buckwheat (*Fagopyrum tataricum*), a pseudocereal, is rich in protein, minerals, and unique bioactive substances, especially phenolic compounds (such as rutin and quercetin) [1]. It exhibits remarkable physiological functions, including antioxidant, anti-inflammatory, as well as blood sugar- and lipid-lowering properties, demonstrating substantial potential in functional foods and pharmaceuticals [2]. In general, buckwheat grains are dehulled before further processing. Hence, Tartary buckwheat hulls (TBH) are generally considered a by-product or waste, resulting in low resource utilization during the processing. Notably, most studies have shown that TBH is also abundant in phenolic compounds (479.65 mg/100 g), including phenolic acids (e.g., protocatechuic acid, ferulic acid, p-coumaric acid), flavonoids (e.g., rutin, quercetin), and anthocyanins, potentially exceeding the levels found in the kernels (339.34 mg/100 g) [3,4,5]. This suggests that the hulls could serve as a valuable natural source of phenolics. Nevertheless, research on the extraction of phenolics from Tartary buckwheat has primarily focused on grains and bran [1,6,7]. TBH, the outer protective layer of the grain, is composed of multiple cell wall layers with a total fiber content exceeding 50% [8]. Bound phenolics account for approximately 16% of the total phenolic content in TBH, which is significantly higher than the proportion found in other parts of the plant [9]. However, effective methods for releasing these cell wall-bound polyphenols remain a significant technical challenge. Therefore, developing efficient extraction techniques to liberate polyphenols from TBH is a primary focus of this study.

Conventional extraction methods (such as Soxhlet extraction and solvent extraction) have certain limitations, including low efficiency, degradation of heat-sensitive phenolics at high temperatures, and significant consumption of organic solvents [10]. Therefore, it is urgent to explore new extraction methods to replace traditional ones. Enzymatic hydrolysis is an environmentally friendly technique that offers mild conditions and reduced solvent consumption [11]. It works by specifically hydrolyzing plant cell wall components (e.g., cellulose, hemicellulose, and pectin), disrupting the dense structure and promoting the release of intracellular phenolics [12]. Nevertheless, the single enzymatic process is slow, and enzyme-substrate contact efficiency may be limited. To overcome these limitations, various green and efficient technologies have been applied to assist extraction, including ultrasound [12], microwave [10], and pulsed electric fields [13]. Among them, ultrasound-assisted extraction has garnered considerable attention due to its cavitation effect, which disrupts cellular membranes and enhances mass transfer across cell walls [12]. Ultrasound treatment can rapidly disrupt the physical structure of the husk, increasing enzyme-substrate contact area and permeability, thereby facilitating enzymatic reactions [14]. In summary, ultrasound-assisted enzymatic extraction combines high efficiency, low energy consumption, and excellent scalability. Its mild operational conditions help maintain the stability of bioactive compounds, rendering it particularly well-suited for the industrial production of functional ingredients. So far, ultrasound-assisted enzymatic hydrolysis has not been applied to extract phenolics from TBH. Therefore, in this study, ultrasound-assisted cellulase treatment was performed to enhance the phenolic extraction process.

On the other hand, extraction is a multiscale physicochemical phenomenon involving coupled transport mechanisms. However, due to the microscopic scale of ultrasonic cavitation and the instantaneous nature of ultrasound, it is difficult to accurately characterize the dynamic process using experimental methods [15]. Phenomenological modeling is a virtual tool for exploring mass transfer mechanisms involved in extraction. Current research generally relies on empirical and semi-empirical formulas, such as first-order and second-order kinetic models, as well as Sovová’s model, to fit extraction data [16]. However, the physical significance of such methodologies is limited and cannot fully analyze the dynamic spatial-temporal evolution characteristics during ultrasound-enhanced extraction. At present, some mathematical modeling methods have been applied and established in the kinetic analysis of component extraction to explain the mass transfer mechanism of solid-liquid extraction of natural raw materials, including Fick’s law, rate law, Peleg’s empirical model, and other two-parametric empirical models [17]. Among them, Fick’s law describes the macroscopic principles of the material diffusion process and is applied to the phenolic extraction process of walnut pellicle [16], chokeberry [18], pomegranate skin [19], grape marc [20], and apple skin [21]. Up to now, its application in the ultrasound-assisted extraction of phenolics from Tartary buckwheat hulls is unclear. Additionally, the adaptive neuro-fuzzy inference system (ANFIS) is considered a breakthrough computational intelligence framework, thereby being widely used in engineering and biological process modeling [22]. ANFIS combines the advantages of neural network (NN) and fuzzy inference system (FIS), demonstrating more efficient training speed, more powerful learning algorithms, and superior prediction performance than traditional neural networks [18]. In the ultrasound-enhanced extraction process, the extraction rate is affected by various ultrasonic parameters (such as power levels and sonication time) and non-ultrasonic parameters (such as environmental temperature and solvent). Specifically, within a certain range, extraction efficiency is positively correlated with ultrasound time, power levels, and environmental temperature. Moreover, choosing a suitable solvent according to the characteristics of the target compounds greatly improves extraction performance [15]. Existing statistical and empirical models do not consistently and accurately characterize the complex associations between all these parameters and target variables. Therefore, applying ANFIS to analyze the interaction between these factors and extraction rates has significant advantages. Notably, ANFIS has yet to be applied to model the process of ultrasound-enhanced extraction of TBH phenolic.

Based on the abovementioned phenomenon, the current study was focused on the effect of various extraction parameters, including enzyme concentration, sonication time and temperature, on extraction efficiency and the mass transfer mechanism of phenolics from TBH. For this, the diffusion model based on Fick’s law was applied to compare the effective diffusion coefficient during ultrasound-assisted enzymatic extraction of phenolics from TBH. Then, ANFIS was employed to predict the influences of various extraction parameters on the extraction yield of phenolics. Additionally, phenolics extracted from TBH were identified and quantified by high-performance liquid chromatography (HPLC) to further understand the effect of different extraction parameters on the individual phenolic compounds.

## 2. Materials and Methods

### 2.1. Preparation of Tartary Buckwheat Hull

A commercial Tartary buckwheat grain with hull purchased by a local supermarket was used for phenolic extraction. The place of origin is Liangshan Yi Autonomous Prefecture in Sichuan Province. Due to its unique climate, the quality of bitter buckwheat in this area is relatively high. Whole Tartary buckwheat grains were ground at room temperature using a grinder for 3 min, and then sifted through an 80-mesh sieve. Tartary buckwheat hulls (TBH) (remained on the sieve) were collected, ground again for 8 min, sifted through an 80-mesh screen, and stored at 4 °C until further analysis. The sieve residue, identified as Tartary buckwheat hull (TBH) particles, was collected and stored at 4 °C until further analysis.

### 2.2. Particle Size Distribution of TBH

**Figure A1:**
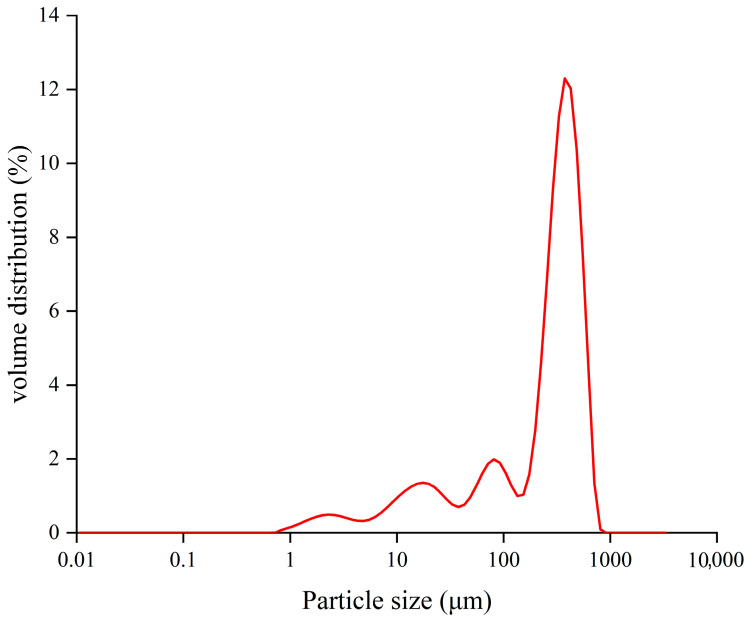
Particle size distribution diagram of TBH particles.

The particle size of the TBH granules was measured using the laser diffraction particle size analyzer (Mastersizer 3000, Malvern Panalytical Inc., Malvern, Worcestershire, UK). As a result, the average particle size distribution (D_[2,3]_) of the TBH particles was 49.00 ± 0.50 μm (Figure A1).

### 2.3. Extraction Protocol of Phenolics

To compare the extraction efficiency of TBH phenolics using different methods, three extraction techniques were employed. The traditional magnetic stirring extraction (without ultrasound or enzyme treatment) was used as the negative control to assess the baseline extraction efficiency. The ultrasonic extraction served as the positive control when evaluating the effect of enzymatic pretreatment. The ultrasound-assisted enzymatic extraction (with different cellulase concentrations) was used to assess the enhancement effect of enzymatic treatment compared to the positive control. A stock solution of TBH at a solid-to-liquid ratio of 1:10 was prepared by dispersing the TBH powder in deionized water (60 mL). The mixture was adjusted to pH 5.0 using 1 M HCl, which corresponds to the optimal pH for cellulase activity. Subsequently, the mixtures were incubated in a 50 °C water bath for 20 min to allow the enzymatic reaction, followed by heat treatment at 80 °C for 2 min to inactivate the enzyme. Although this step was primarily designed for the enzymatic pretreatment in the ultrasonic-assisted enzymatic extraction group, the same procedure was applied to the other extraction methods to ensure strict control of variables and maintain comparability among experimental results.

According to literature reports, the yield tends to decrease at temperatures above 50 °C [23,24]. Therefore, magnetic stirring (at 800 rpm) and ultrasonication at 20 kHz using an ultrasonic processor (20 kHz, XO-1000D, Xianou Instrument Manufacturing Co., Ltd., Guangzhou, Guangdong, China) equipped with a 10 mm diameter sonotrode probe was used at an actual power of 41.88 W were carried out at 40 °C and 50 °C, respectively. At each temperature, samples were collected at the same time intervals to determine the total phenolic content, allowing a direct comparison of the extraction efficiencies between the two methods. The total extraction time was determined through preliminary experiments, and sampling time points were selected based on the faster extraction in the early stage and slower extraction in the later stage.

The ultrasonic-assisted enzymatic extraction method involved an enzymatic pretreatment step prior to ultrasonic extraction. Given previous reports indicating that excessively high enzyme concentrations may reduce extraction efficiency and considering the economic cost of enzyme usage, this study selected relatively low cellulase (derived from *Aspergillus niger*) dosages (0.5% and 1%) [25]. Apart from this, the extraction temperatures and sampling time points of the extract were kept consistent with those of the ultrasonic extraction group to ensure comparability of experimental conditions.

### 2.4. Determination of Total Phenolic Content

The total phenolic content of TBH was determined by following the procedure reported by Bhinder, et al. [26] with minor modifications. Briefly, 0.25 mL of the TBH phenolic extract was diluted to 80% of the original concentration, and 0.3 mL of the Folin-Ciocalteu reagent (1 mol/L) was mixed. Then, 10% Na_2_CO_3_ solution (0.75 mL) and deionized water (4 mL) were added to the above mixture and react in the dark at room temperature for 40 min. The absorbance of the solution was measured at a wavelength of 765 nm using a Thermo Scientific Varioskan Flash (Thermo Fisher Scientific, Waltham, MA, USA). A standard solution was prepared using gallic acid, and a calibration curve was obtained using a range of concentrations from 0 to 0.45 g/L. The results were expressed as gallic acid equivalents (GAE) in mg/g of TBH on an as-received basis (as described in Section 2.1). Subsequently, to accurately determine the total phenolic content in TBH particles, the samples underwent two consecutive extractions, and the filtrates were combined for subsequent total phenolic content analysis. The calculated total phenolic content in the TBH granules was determined to be 7.90 ± 0.02 mg GAE/g.

### 2.5. Modeling of Ultrasound-Assisted Extraction of Phenolics

To evaluate the effects of ultrasound-related parameters on mass transfer during ultrasound-assisted enzymatic hydrolysis extraction, a diffusion model based on Fick’s second law was employed to analyze the extraction kinetics of phenolic compounds obtained via ultrasound-assisted enzymatic hydrolysis extraction under varying conditions. The original code for this analysis is provided in Appendix A. Before modeling, fundamental assumptions were established as follows [15,18]:

(a) TBH granules were modeled as spheres with an average diameter of 49.00 μm, containing uniformly distributed phenolic compounds.
(b) Micro-turbulence and cavitation bubbles ensured thorough mixing of the extraction suspension, rendering external mass transfer resistance negligible.
(c) The effective diffusion coefficient ($D_{e}$) maintained constant values during extraction due to insignificant changes in particle size and external temperature.
(d) In the extraction process, the swelling of TBH particles were not considered, and changes in particle size were neglected.
(e) No degradation of phenolic compounds during sonication was considered.
(f) The phenolic concentration at the particle-solvent interface equilibrated with that in the adjacent solvent phase.

Based on these assumptions, TBH phenol mass transfer was modeled by the spherical diffusion governing equation (Equation (1)):

(1) $$\frac{\partial C_{s}}{\partial t}=D_{e}\left(\frac{1}{x^{2}}\frac{\partial }{\partial x}\left(x^{2}\frac{\partial C_{s}}{\partial x}\right)\right)$$

where $C_{s}$ stands for the concentration of the TBH phenolic (g/cm^3^); t indicates extraction time (min); $D_{e}$ represents the effective diffusion coefficient of total phenolic (m^2^/s); x denotes the spherical particle radial coordinate (m).

The initial condition of diffusion model is shown by Equation (2):

(2) $$C_{L}\left(0\right)=0,C_{s}\left(x,\;0\right)=C_{s}^{0}$$

where $C_{L}\;$ stands for total phenolic content in liquid phase (g/mL).

The boundary condition is given in Equations (3) and (4):

(3) $${\frac{\partial C_{s}\left(0,t\right)}{\partial t}}_{x=0}$$

(4) $${-D_{e}S\frac{\partial C_{s}\left(x,t\right)}{\partial x}=V_{L}\frac{dC_{L}\left(t\right)}{dt}}_{x=r}$$

where S stands for contacting area among the extracting solvent and particles (m^2^); $V_{L}$ stands for the volume of suspension (mL); r denotes particle radius (m).

The parabolic partial differential equations were solved numerically via the pdepe function in MATLAB R2010a (The MathWorks, Inc., Natick, MA, USA) under specified initial/boundary conditions. Spatial discretization of equations yielded a system of ordinary differential equations, which was temporally integrated to generate solutions at designated time points. The effective diffusion coefficient was iteratively optimized to minimize the root mean square error (RMSE) between simulated and experimental phenolic contents in TBH granules. Meanwhile, the coefficient of determination (R^2^) and absolute average deviation (AAD) were also utilized to evaluate the accuracy of the diffusion model. These are respectively shown by Equations (5)–(7) below.

(5) $$RMSE(mg/g)=\sqrt{\frac{1}{n}\sum_{i=1}^{n}{\left[C_{s,p}\left(t\right)-C_{s,e}\left(t\right)\right]}^{2}}$$

(6) $$R^{2}=1-\frac{\sum_{i=1}^{n}{\left(C_{i,e}-C_{i,p}\right)}^{2}}{\sum_{i=1}^{n}{\left(C_{i,e}-C_{i,avg}\right)}^{2}}$$

(7) $$AAD\left(\%\right)=\left[\frac{\sum_{i=1}^{n}\left(\left|C_{i,e}-C_{i,p}\right|/C_{i,e}\right)}{n}\right]\times 100$$

where $C_{s,\;p}$ represents total phenolic content from TBH granules predicted by diffusion model (g/cm^3^); $C_{s,e}$ represents total phenolic content from TBH granules acquired through experiments (g/cm^3^); $C_{i,\;e}$ stands for experimentally determined mass of phenolic extracted from TBH granules (mg/g); $C_{i,\;p}$ denotes predicted mass of phenolic extracted from TBH granules (mg/g); $C_{i,\;avg}$ is the average value of the mass of phenolic extracted from TBH granules across all the experimental data.

### 2.6. ANFIS Modeling

To elucidate the interactions among key parameters (enzyme concentration, temperature, and extraction time) and validate the predictive performance of ANFIS for phenolic yield, the ultrasound-assisted enzymatic hydrolysis extraction of phenol from TBH was modeled using ANFIS according to the procedure described by Tao, Wang, Pan, Zhong, Wu, Yang, Han and Zhou [18]. ANFIS integrates the computational power of artificial neural networks (ANN) with the human-like reasoning capabilities of fuzzy inference systems (FIS) [22]. The ANFIS model was designed using MATLAB 2024a and consists of five layers: fuzzification layer (I), rule layer (II), normalization layer (III), defuzzification layer (IV), and output layer (V). The construction process of the ANFIS model is outlined as follows: 48 sets of experimental data were input, with temperature, time, and enzyme concentration as the input variables, and total phenolic content as the output variable. An initial Sugeno-type fuzzy inference system was constructed, and fuzzy rules as well as initial membership function parameters were generated using the grid partitioning method. The ANFIS model was subsequently trained with two algorithms: backpropagation and hybrid (combining forward and backward passes). The model was iteratively trained until the error converged, and the final model was obtained and applied for predictive.

During the modeling process, 48 samples were randomly divided into training and testing subsets at a ratio of 6:4. To avoid overfitting, key input variables and target values were uniformly distributed between the two subsets, preventing the over-concentration of specific features within a single subset. Training was stopped when the RMSE of either the training or testing dataset reached the lowest point. Gaussian (dsigmf) was utilized as the input MF in the fuzzification layer. Three replicate experiments were conducted under identical conditions, with observed natural variation reflected by standard deviations ranging from 0.002 to 0.150. To reduce the influence of outlier data on model accuracy, the mean value of each group was used as input and output data for training the ANFIS model. Based on this approach, the model demonstrated stable performance in capturing data trends, indicating good adaptability to experimental variability.

### 2.7. Quantification of Phenolic Compounds Through HPLC Analysis

To illustrate the major compositions of the extracted TBH phenolic, the raw TBH phenolic extracts were analyzed through HPLC as described by Zhang, et al. [27] with some modifications. HPLC analysis was performed using HPLC system (LC-2010A HT, SHIMADZU (CHINA) Co., Ltd., Shanghai, China) equipped with an Inertsil ODS-3 (4.6 × 250 mm, 5 µm, GL Sciences, Tokyo, Japan), with ultrapure water with 1% acetic acid (A) and methanol with 1% acetic acid (B) as mobile phase. The phenolic acids and flavonoids elution linear gradient for B solvent was from 10 to 26% for 10 min, from 26 to 40% for 15 min, from 40 to 65% for 20 min, and then reached to 95% at 45 min and held for 10 min, 95 to 10% for 3 min, and reached to 10% at 65 min. During elution, the column temperature was maintained at 25 °C, and the injection volume was 20 μL. The flow rate was set at 0.6 mL/min. Phenolic compounds were detected at 280 nm and 350 nm.

### 2.8. Statistical Analysis

All experiments were replicated three times. The results were expressed in mean ± standard deviation. Statistical significance was determined by one-way ANOVA followed by Duncan’s test using SPSS software (SPSS Inc., version 22.0, Armonk, New York, NY, USA). *p* < 0.05 indicating a significant difference. Results were plotted graphically using Origin 2021 scientific software.

## 3. Results

### 3.1. Comparative Analysis of the Kinetic Behavior of Phenolic Yields Under Different Extraction Conditions

**Figure 1:**
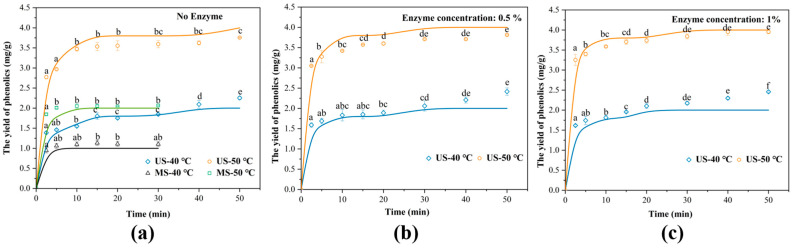
The extraction yields of total phenolics from TBH powder under different enzyme concentrations. (**a**) No Enzyme; (**b**) 0.5%; (**c**) 1%. The error bars show ± standard deviation values. Different letters indicated significant differences (*p* < 0.05). Note: Different symbols represent the experimental data, and solid lines represent the simulated kinetic curves obtained from the diffusion model. US stands for ultrasonic extraction, and MS stands for magnetic stirring extraction.

The efficiency of ultrasonic extraction of phenolics from TBH particles was assessed by comparison with conventional magnetic stirring extraction at 40 °C and 50 °C (Figure 1a). Within the 30 min extraction period, both methods exhibited a rapid initial increase in extraction yield, followed by a gradual plateau. However, during the initial rapid extraction phase, ultrasonic extraction exhibited superior performance compared to magnetic stirring. Specifically, after 10 min, the extraction yields of phenolics by ultrasound reached 1.55 ± 0.02 mg/g at 40 °C and 3.47 ± 0.05 mg/g at 50 °C, which were 1.42 and 1.69 times higher, respectively, than those obtained with magnetic stirring. Throughout the entire extraction period, ultrasonic extraction consistently yielded higher amounts of phenolics from TBH particles compared to magnetic stirring at the same temperature. Moreover, for both extraction methods, the yield at 50 °C was consistently higher than that at 40 °C. The results clearly demonstrate the superiority of ultrasonic extraction over the conventional method. Elevated temperatures further improved the extraction efficiency of phenolics from TBH, likely due to enhanced diffusivity and solubility [28]. However, in plant-based matrices, the cell wall serves as a major barrier to the extraction of polyphenols. Specifically, a portion of polyphenols can form stable complexes with cell wall components such as cellulose, hemicellulose, and polysaccharides, thereby increasing the difficulty of their extraction or release. Meanwhile, polyphenols that do not bind to the cell wall may still be entrapped within the intracellular space, preventing their free diffusion. In addition, the compact arrangement of plant cells and the small pore size of the cell wall further limit the interaction between solvents and polyphenols [29]. Therefore, in this study, ultrasound-assisted enzymatic extraction was employed to enhance the release of polyphenols from TBH.

The effects of enzyme concentration and extraction temperature on the extraction kinetics of total phenolics from TBH are shown in Figure 1. Temperature remained the dominant factor affecting ultrasonic extraction efficiency [30,31,32]. The extraction kinetics typically exhibit two phases: a washing phase and a diffusion phase, corresponding to the rapid and slow extraction stages, respectively. As shown in the figure, nearly 70% of the phenolics were extracted within the first 10 min under all tested conditions [33]. At this stage, the extracted polyphenols are mainly those present in TBH in free or weakly bound forms, which are distributed in the cell sap or on the cell surface. These compounds can be rapidly desorbed by the solvent without the need to disrupt the cell wall, resulting in minimal mass transfer resistance and a characteristic fast desorption phase in the extraction kinetics. With increasing cellulase concentration, the final extraction yield of total phenolics showed no significant increase. However, during the initial rapid extraction phase, ultrasound following cellulase pretreatment significantly improved extraction efficiency and reduced extraction time. Specifically, after 5 min of ultrasonic extraction, total phenolic yields of untreated samples were 1.46 ± 0.02 mg/g (40 °C) and 2.97 ± 0.02 mg/g (50 °C). In contrast, samples pretreated with 1% cellulase achieved higher yields of 1.74 ± 0.10 mg/g (40 °C) and 3.40 ± 0.05 mg/g (50 °C). This may be attributed to the action of cellulase, which degrades the cellulose components of the cell wall, loosening the originally compact structure of TBH. As a result, the porosity increases and the local wall thickness decreases, significantly reducing the barriers to solvent penetration and polyphenol release, thereby enhancing the efficiency of the washing phase during ultrasonic-assisted extraction [34].

### 3.2. Analysis of Mass Transfer Dynamic Parameters Based on Diffusion Model

To further quantify the differences in mass transfer efficiency, numerical simulations based on Fick’s law were used to fit the extraction process. The key kinetic parameters obtained are shown in Table 1.

Figure 1 presents the experimental data and mode-predicted extraction rates of phenolics under different extraction conditions. According to Table 1, the R^2^ values fluctuate around 0.90, and the AAD values range from 3.031% to 10.075%. Under all conditions, the RMSE values of the dynamic curves remained low. Therefore, despite slight deviations at certain points, the diffusion model reliably reflects the extraction process of phenolics from TBH particles. According to the data in Table 1, ultrasound-assisted extraction significantly increased the effective diffusion coefficients ($D_{e}$) compared to magnetic stirring. Specifically, at 40 °C, the increase in $D_{e}$ from 5.70 × 10^−7^ to 9.15 × 10^−7^ m^2^/s facilitates the migration of phenolics from TBH particles into the surrounding solution. This is consistent with the findings of Hebin Xu et al. [35]. Cellulose acts on the cell wall of TBH, making its structure loose. Therefore, ultrasound-assisted enzyme extraction further increases the $D_{e}$. At 50 °C, treatment with 1% cellulase raised the $D_{e}$ value from 4.30 × 10^−7^ to 6.09 × 10^−7^ m^2^/s. However, with increasing temperature, the effective diffusion coefficient ($D_{e}$) exhibited a decreasing trend. For example, in the ultrasound group without enzymatic pretreatment, $D_{e}$ decreased from 9.15 × 10^−7^ m^2^/s to 4.30 × 10^−7^ m^2^/s as the extraction temperature increased from 40 °C to 50 °C. Similarly, Yang Tao et al. reported that during ultrasound-assisted extraction of phenolic compounds from grape pomace, the highest $D_{e}$ of 4.92 × 10^−11^ m^2^/s was observed at 40 °C, while it dropped to 4.51 × 10^−11^ m^2^/s at 50 °C [20]. Hojnik et al. also reported that phenolic compounds exhibited higher effective diffusion rates at lower temperatures during mechanical stirring extraction [35]. In their study, when the temperature increased from 40 °C to 60 °C, $D_{e}$ decreased from 2.175 × 10^−8^ cm^2^/s to 1.503 × 10^−8^ cm^2^/s, although the total extraction yield continued to rise. Although these studies did not provide an explanation for this phenomenon, the present study hypothesizes that it may be related to heat-induced re-adsorption of polyphenolic compounds at elevated temperatures. Renard et al. reported that following tissue fragmentation or heat treatment, anthocyanins spontaneously bind to cell wall polysaccharides, with pectin being the main binding target [36]. This suggests that even under moderate temperatures, where no obvious structural collapse occurs, phenolic molecules may still be re-adsorbed or cross-linked within the tissue. Such interactions can obstruct internal diffusion pathways, reduce $D_{e}$, and hinder the migration of phenolics from the interior to the particle surface. Although the effective diffusion coefficient decreased with increasing temperature, the overall extraction yield still improved due to the combined effects of reduced solvent viscosity and surface tension, enhanced permeability, and increased solubility of polyphenols at higher temperatures [37]. The model relies on simplified assumptions regarding particle geometry, which may limit its ability to fully represent the heterogeneous and complex microstructure of the material. Such simplifications are common in numerical simulations to balance computational efficiency and model tractability. While this introduces inherent limitations, the experimental results remain robust and provide valuable insights into the extraction process.

### 3.3. Phenolic Concentration Gradient Within TBH Particles Under Different Ultrasonic-Assisted Enzymatic Extraction Conditions

**Figure 2:**
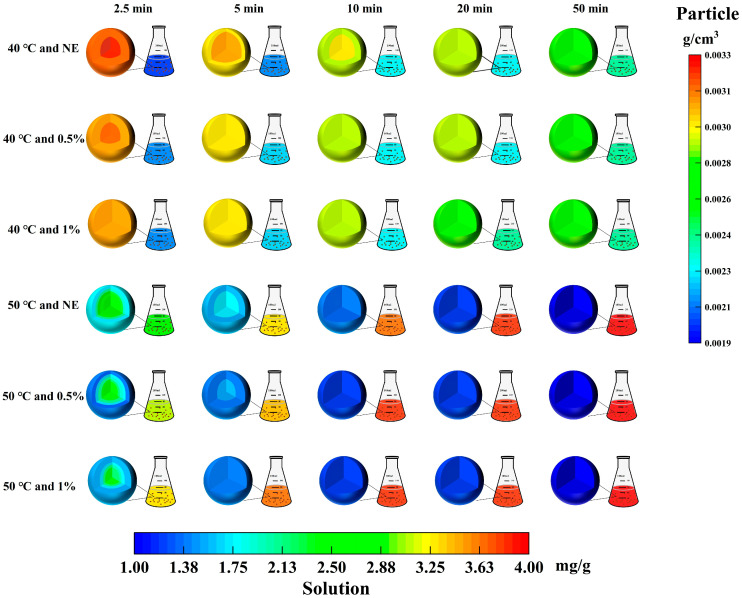
Variations in phenolic content in TBH particles and the surrounding solution under different ultrasound-assisted enzymatic conditions (temperature (°C) and enzyme concentration (%)).

The data presented in Figure 2 were obtained through numerical simulation based on Fick’s second law. The simulation was conducted assuming spherical particles and describes the variation in polyphenol concentration at different time points and spatial positions during the extraction process. The 7.90 ± 0.02 mg GAE/g value refers to the total extractable polyphenol content within the particles.

To better elucidate the microscopic process of ultrasound-assisted enzymatic extraction of phenolics, representative time points were selected for each condition, corresponding to the rapid extraction stage, the slow extraction stage, and the point of maximum yield. Spatial distribution maps of phenolic concentration within TBH particles and the surrounding solution were constructed at selected time points under different treatment conditions (Figure 2). The results showed that without cellulase pretreatment, phenolic distribution within TBH particles during ultrasound extraction at 40 °C remained uneven in the first 10 min, with uniformity achieved only after 20 min. In contrast, 0.5% cellulase pretreatment resulted in uniform distribution by 5 min, while increasing the concentration to 1% led to uniformity as early as 2.5 min. A similar trend was observed at 50 °C, where higher cellulase concentrations accelerated the attainment of homogeneous phenolic distribution.

It is worth noting that at the same enzyme concentration and extraction time, higher ultrasound temperature results in more pronounced phenolic concentration gradients and stratification within TBH particles. For example, at 2.5 min and 0.5% cellulase, the phenolic concentration ranged from 0.0033 g/cm^3^ (r = 0 cm) to 0.0032 g/cm^3^ (r = 0.0245 cm) at 40 °C, but decreased to 0.0026–0.0021 g/cm^3^ over the same radius at 50 °C. These results suggest that although elevating the temperature from 40 °C to 50 °C may impede the internal diffusion of phenolics within the solid matrix, a greater amount of phenolics is nevertheless rapidly released into the solvent phase during the initial extraction stage. A likely explanation is that high temperature accelerates the release of outer phenolics, enhances compound solubility, and reduces solvent viscosity [38]. However, the internal structure remains largely intact, impeding the migration of inner-layer phenolics and causing a slower diffusion rate compared to the outer layer. These findings suggest that phenolic migration during extraction is jointly governed by thermal diffusion and structural barriers. As shown in Figure 2, cellulase pretreatment effectively disrupts these barriers.

In general, increasing temperature alone cannot substitute for structural pretreatment, and may even exacerbate uneven mass transfer caused by incomplete structural degradation. Therefore, rational optimization of enzymatic hydrolysis conditions, combined with temperature elevation based on sufficient structural pretreatment, is essential for achieving efficient and uniform phenolic release from plant materials [39].

### 3.4. ANFIS Modeling Under Different Enzyme-Assisted Ultrasound Conditions

To accurately predict and regulate the phenolic extraction efficiency under multi-factor conditions, an adaptive neural fuzzy inference system (ANFIS) was employed. ANFIS integrates the interpretability of fuzzy logic with the learning capability of neural networks, effectively addressing the limitations of traditional Response Surface Methodology (RSM) and Artificial Neural Networks (ANN). RSM is a regression-based statistical optimization tool suitable for linear or near-linear systems, but it struggles with complex nonlinear relationships and variable interactions. ANN can handle nonlinear problems well but often lacks transparency. In contrast, ANFIS offers high predictive accuracy while maintaining a certain level of interpretability, making it more suitable for modeling and optimizing complex, multivariable nonlinear systems. Multiple reports indicated that its predictive performance is surpasses that of response surface methodology (RSM) [40] and artificial neural networks (ANN) [41].

**Figure 3:**
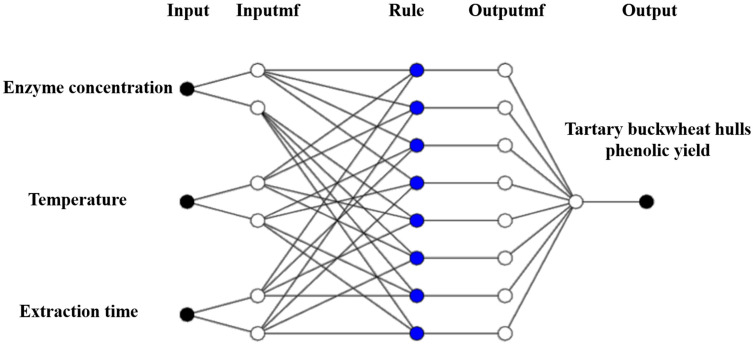
Architecture of the ANFIS model for predicting total phenolic yield during ultrasound-assisted enzymatic extraction from TBH particles.

**Figure 4:**
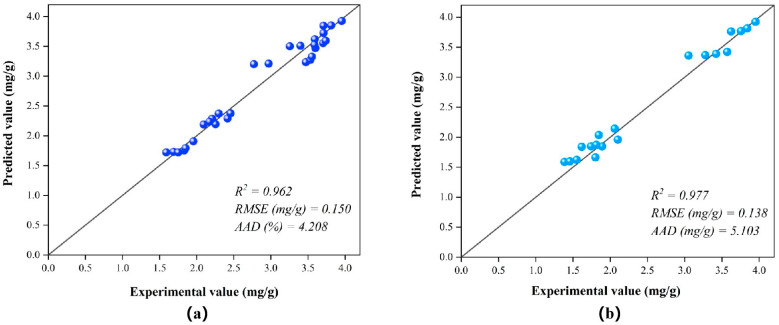
Predicted versus experimental values of total phenolic yield and statistical evaluation of the developed ANFIS model. (**a**) Training phase; (**b**) Testing phase.

**Figure 5:**
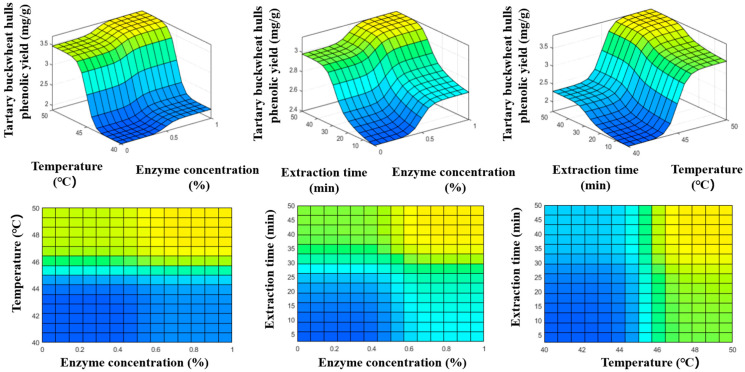
2D and 3D surface plots about the effects of ultrasound-assisted enzymatic extraction conditions (temperature, enzymatic concentration, and extraction time) on total phenolic yield generated by constructed ANFIS model. ‘Blue’ from the ANFIS tool color map was selected. The color gradient represents the phenolic yield, with blue corresponding to the lowest yield and yellow to the highest.

The structure of the constructed ANFIS model is shown in Figure 3. The input variables include temperature, enzyme concentration, and extraction time. Each input variable was assigned two membership functions. After comparing the training errors of models developed using different membership functions, the difference-Sigmoid function (dsigmf) was selected for all input variables. The dsigmf function can effectively simulate the fuzzy feature of “variables being optimal within a specific interval” and better reflect the nonlinear interval response characteristics under actual extraction conditions [42]. Subsequently, the model was trained using a hybrid algorithm with 30 iteration cycles. As shown in Figure 4, the RMSE values of the ANFIS model formed during the training and testing phases of total phenol production are 0.150 (mg/g) and 0.138 (mg/g), respectively. For the same response sequence, the R^2^ values all exceeded 0.96. The higher R^2^ value and lower RMSE value indicate that the ANFIS model successfully predicted total phenol production within the selected experimental range. The effects of ultrasound parameters were visualized using 2D and 3D surface plots generated by the ANFIS model (Figure 5). 

As shown in Figure 5, the synergistic effect of temperature and enzyme concentration on phenolic yield is most significant. Overall, the phenolic yield increases with temperature and enzyme concentration, especially in the temperature range of 46–50 °C and enzyme concentration above 0.6%, where the extraction rate reaches its maximum value, forming a relatively flat high-value interval. This is consistent with the interval-optimal response fitted by the dsigmf fuzzy membership function, indicating that the extraction process is not simply a unimodal optimum, but rather involves a relatively stable and efficient range of conditions. Through the analysis of the influence of enzyme concentration and extraction time on the total phenol yield, it can be concluded that when the enzyme concentration reaches 0.6% or more, the yield rapidly increases in the early stage of extraction, indicating that cellulase assists in breaking down the cell wall and plays a key role in the mass transfer process. Moreover, increasing the ultrasonic extraction time can further break down the structural barrier and improve the phenolic yield. The interaction between temperature and extraction time is manifested as higher temperatures leading to higher levels of phenolic yield in the same amount of time. In the 2D graph, it can be seen that extending the extraction time within 40–45 °C did not result in a significant increase in the extraction rate, indicating that temperature is the main factor affecting the release of phenolics from TBH particles into the solvent.

Based on the interaction results of three variables, it can be concluded that the phenolic extraction process is influenced by multiple factors. Enzyme concentration enhances cell wall degradation and facilitates the rapid extraction stage. Temperature mainly affects the diffusion rate, while ultrasonic extraction time ensures the continuous release of phenolics. The ANFIS model effectively simulates the relationships between nonlinear variables, providing a theoretical basis for extraction process optimization. Although the ANFIS model demonstrates strong performance in capturing complex nonlinear relationships, it has several limitations: high computational cost and rule explosion with increasing input variables, reduced interpretability with more fuzzy rules, and limited extrapolation capability beyond the trained data range. Such issues have been documented in prior studies [43]. Future work could include dimensionality reduction, meta-heuristic optimization, or cross-validation to improve the model’s robustness and generalizability.

### 3.5. Identification and Content Analysis of Main Phenolic Components in Phenolic Mixtures

**Figure 6:**
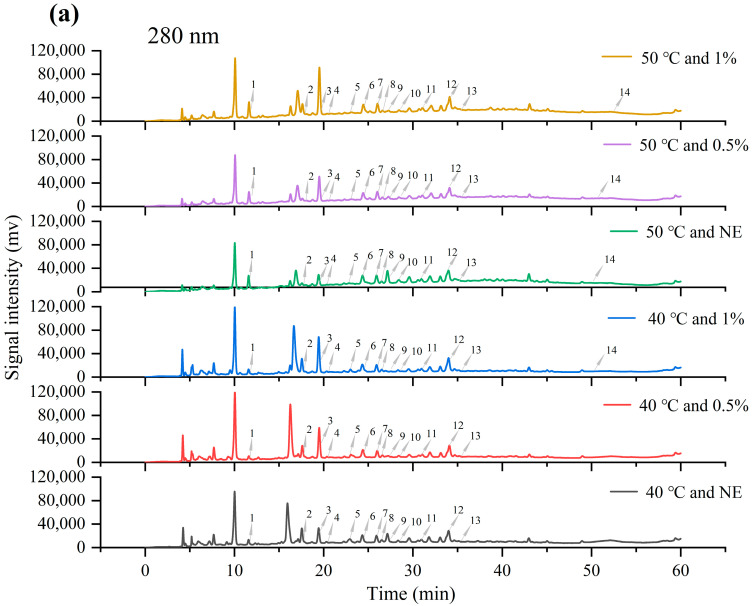

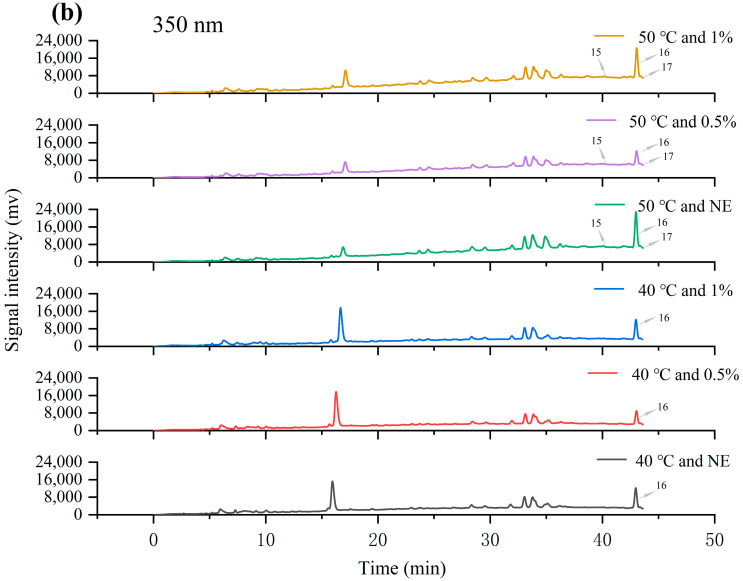
HPLC spectra of phenolic extracts obtained from different ultrasound-assisted enzymatic conditions (temperature (°C) and enzyme concentration (%)) at 280 nm (**a**) and 350 nm (**b**). 1—Gallic acid, 2—Protocatechuic acid, 3—Catechin, 4—Procyanidin B2, 5—Chlorogenic acid, 6—p-Hydroxybenzoic acid, 7—Epicatechin, 8—Homovanillic acid, 9—Caffeic acid, 10—Syringic acid, 11—Phloretic acid, 12—p-Coumaric acid, 13—Ferulic acid, 14—Phloretin, 15—Rutin, 16—Myricetin, 17—Quercetin-3-O-rutinoside. Note: Due to the extremely low abundance of certain compounds, their peaks are not visible in the chromatogram. The labeled positions indicate the retention times of the corresponding standards and are provided for reference only. Quantitative concentrations of these compounds are provided in Table 2.

**Figure 7:**
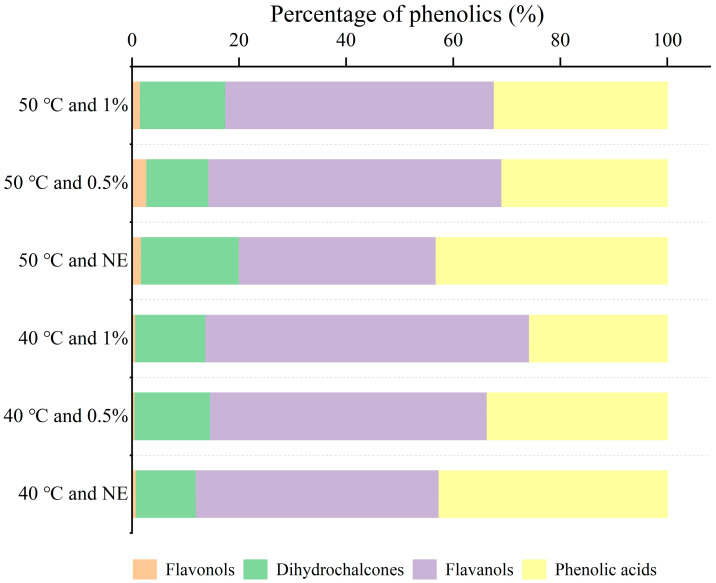
Proportion of phenols of different categories under different ultrasound assisted enzymatic hydrolysis extraction conditions (temperature (°C) and enzyme concentration (%)).

After characterizing the macroscopic mass transfer kinetics of phenolics under different processing conditions, this study conducted qualitative and quantitative analyses of phenolic components in the extract to further assess compositional changes under various extraction methods. High-performance liquid chromatography (HPLC), combined with the retention times and calibration curves of known phenolic standards, was used to identify and quantify the main phenolic compounds, and the influence of mass transfer behavior on product composition was supplemented at the component level. Figure 6 shows the chromatographic profiles of the extract under various ultrasound-assisted enzymatic extraction conditions, identifying a total of 17 phenolic compounds. Table 2 provides a detailed display of the peak time and specific content of these 17 substances. According to Table 2, most of the components in the mixture belong to phenolic acids, accounting for 9 out of 17 phenolic substances. Among phenolic acids, homovanillic acid, chlorogenic acid, and protocatechuic acid have the highest content, with extraction amounts exceeding 100 μg/g under most conditions. The content of catechins in flavanols is the highest, and except for the extraction condition of no cellulase usage at 50 °C, the extraction amount under other conditions is above 300 μg/g. The consistently high contents of the above compounds under various extraction conditions suggest that they exist in TBH primarily in a relatively free or easily releasable form. Catechin content showed a positive correlation with enzyme concentration, especially at 50 °C, where it increased from 44.08 ± 3.45 μg/g to 855.66 ± 10.93 μg/g. This suggests that catechin may exist in a bound form within the TBH particles. This is consistent with the research findings of Baruah et al. [44]. Lutin and quercetin in flavonols did not show up at 40 °C but were detected at 50 °C. Like catechins, the extraction amount increased with the increase of cellulase dosage [45]. This phenomenon may be attributed to rutin being bound to cell wall components via glycosidic or ester linkages, forming a relatively stable conjugated state. Enzymatic hydrolysis is needed to break the cell wall structure and noncovalent binding force before they can be released into the solution. In addition, an increase in temperature can enhance its solubility in the aqueous phase, thereby increasing its detected concentration. By using Figure 7 bar chart, the changes in the proportion of various phenolic substances can be more intuitively observed. The proportion of flavanols is always the highest, and with the increase of enzyme concentration, the proportion of dihydrochalcones and flavanols gradually increases.

**Figure 8:**
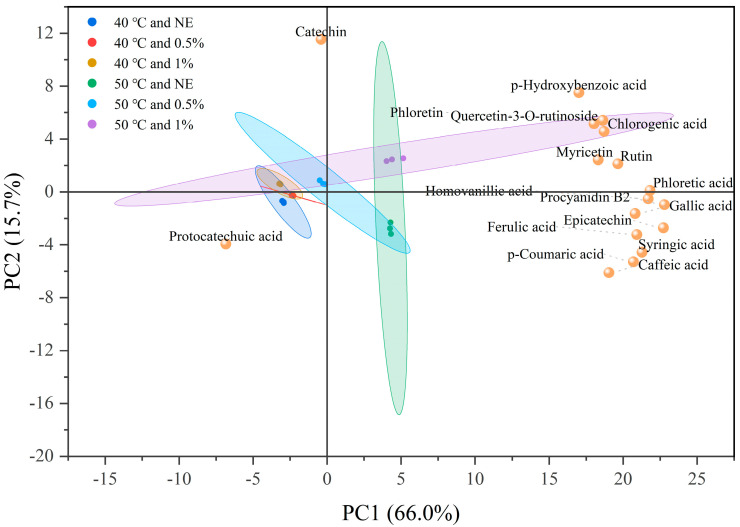
PCA biplot illustrating the scores of extraction conditions and the loadings of characteristic compounds on the principal components. Ovals with the same color as the experimental conditions represent the confidence intervals of the sample points for the corresponding experimental conditions.

PCA was performed based on the data presented in Table 2, with six different extraction conditions (performed in triplicate) as samples and the contents of 17 compounds as variables. Through dimensionality reduction, the analysis aimed to explore the differences among samples and the relationships between compounds and extraction conditions. Figure 8 presents the sample scores and the loadings of the 17 compounds, clearly illustrating the influence of different extraction conditions on the extraction efficiency of each compound. PC1 and PC2 explain 66.0% and 15.7% of the total sample variance, respectively, and together explain 81.7% of the information between samples. The analysis is representative. The main loadings of PC1 were procyanidin B2, phloretic acid, and epicatechin, while the main contributors to PC2 were catechin, p-hydroxybenzoic acid, and caffeic acid. In this study, the interpretation of the PCA plot is primarily based on the spatial relationship between sample scores and variable loadings. Although there is some overlap in the confidence ellipses of different treatment groups, the alignment of variables and samples along the same principal component axis can still be used as an indication of their potential association. From the spatial distribution trend of the samples, it can be seen that the 40 °C treatment group and the 50 °C treatment group are located on both sides of the positive and negative axes of PC1. The catechins and protocatechuic acid in the left quadrant were close to the 40 °C and NE treatment group, indicating that they are more likely to exist in a natural free state in TBH particles, and the extraction effect is better under mild treatment conditions. Rutin, myricetin, and quercetin-3-O-rutinoside in flavonols are all located in the upper right quadrant and close to the 50 °C and 1% treatment group, indicating that these components have better extraction effects under high enzyme concentration pretreatment and high temperature, and their structures are closely bound to the cell wall; And the lower right quadrant contains multiple phenolic acids that are similar to the 50 °C and NE treatment group, indicating that these components can be well released during extraction by simply heating up.

Based on the above data analysis, it can be concluded that different extraction conditions significantly affect the composition and distribution of phenolic compounds in Tartary buckwheat hulls, providing theoretical support for optimizing the plant phenolic extraction process.

### 3.6. Construction of Mass Transfer Mechanism and Structural Response of Phenolics in TBH Particles

**Figure 9:**
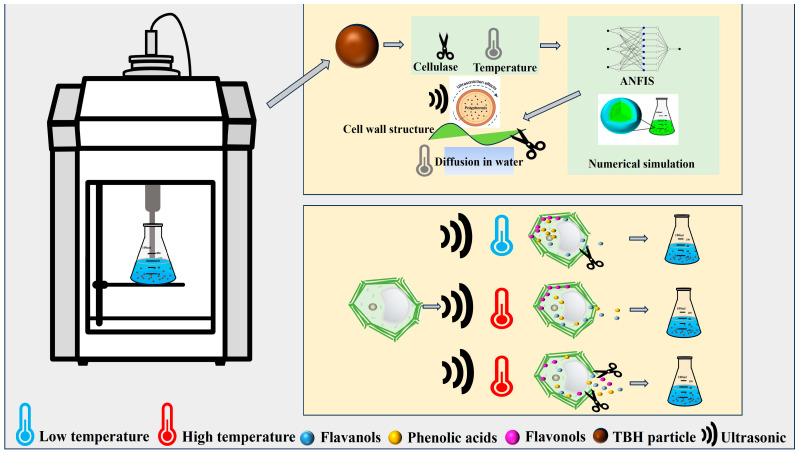
Mass transfer mechanism and structural response diagram of phenolics in TBH particles.

As shown in Figure 9, a mass transfer mechanism diagram based on the combination of “model structure function” was further constructed to reveal the intrinsic mechanism of phenolics migration behavior in Tartary buckwheat shell under ultrasound assisted enzymatic hydrolysis from the perspective of cell structure destruction process and different phenolic structural characteristics. Overall, the synergistic effect of ultrasound induced microjet and shear stress with enzymatic hydrolysis on cell wall structure has greatly improved the efficiency of the rapid extraction stage; Although increasing temperature reduces the effective diffusion coefficient inside the particles, indicating a certain hindrance to the mass transfer process, it can improve solvent performance and enhance the solubility of polyphenols in the solvent, thus leading to a rising trend in the total extraction rate. A series of synergistic effects have constructed a continuous mass transfer channel from cell wall penetration to intracellular diffusion and release into solution. In addition, the impact of ultrasound-assisted enzymatic hydrolysis extraction varies for different types of phenolic substances. Although small molecules, flavanols such as catechin and epicatechin bind tightly within the cell and require strong cavitation or short-range hydrolysis assistance. Phenolic acids (such as p-coumaric acid and ferulic acid) often bind to the cell wall in the form of ester bonds. High temperatures can promote ester bond hydrolysis and enhance the release of bound phenolic acids. Therefore, using temperature drive can effectively promote the rapid diffusion of phenolic acids to the extracellular space. Flavonoids such as rutin and quercetin-3-O-rutinoside are mostly present in bound form on the cell wall structure. Based on ultrasound, mass transfer still relies on enzymatic cleavage and temperature-induced release channel opening, exhibiting high sensitivity to temperature and enzyme synergy. Therefore, the extraction of different phenolics can adopt different focuses, which can not only improve the extraction rate but also greatly save costs.

## 4. Conclusions

This study employed ultrasound-assisted enzymatic extraction to obtain polyphenols from Tartary buckwheat hulls. Compared with conventional extraction methods, the polyphenol yield increased by 91.3%, reaching 396 mg/100 g. By integrating experimental results with numerical simulation based on Fick’s law, a positive correlation was observed between enzyme concentration and the effective diffusion coefficient ($D_{e}$) under identical temperature conditions. Additionally, the adaptive neuro-fuzzy inference system (ANFIS) accurately predicted the extraction efficiency under different temperatures, ultrasonic durations, and enzyme concentrations, demonstrating its high predictive performance and providing a powerful tool for multi-parameter optimization. Finally, catechin and epicatechin were identified as the major components in the extract using HPLC. High temperatures can promote ester bond hydrolysis and enhance the release of bound phenolic acids. At 50 °C, the extraction of flavonoids such as rutin increased with rising enzyme concentration.

Overall, this study employed a water-based, environmentally friendly extraction strategy that significantly enhanced polyphenol extraction rate from TBH while reducing energy and time consumption, demonstrating considerable potential for industrial application. Meanwhile, the polyphenol extract from TBH can be used as a natural antioxidant, with potential applications in extending product shelf life and enhancing nutritional functions. In addition, future work may build on the numerical simulation and ANFIS models established in this study to further explore the process adaptability of ultrasound-assisted enzymatic extraction at pilot scale and broaden the scope of application to other plant-based residues

## Figures and Tables

**Table 1:** Estimated effective diffusion coefficients ($D_{e}$) of total phenolics under different extraction conditions and the goodness of fit of the diffusion model.

Temperature (°C)	Extraction Method	Enzyme Concentration (%)	$D_{e}$ (m^2^/s)	R^2^	RMSE (mg/g)	AAD (%)
40	US	NE	9.15 × 10^−7^	0.963	0.115	5.229
		0.5	1.86 × 10^−6^	0.917	0.175	7.025
		1	2.00 × 10^−6^	0.880	0.219	9.346
	MS	0	5.70 × 10^−7^	0.918	0.102	10.075
50	US	NE	4.30 × 10^−7^	0.972	0.201	6.151
		0.5	5.01 × 10^−7^	0.966	0.225	6.134
		1	6.09 × 10^−7^	0.990	0.121	3.031
	MS	0	3.01 × 10^−7^	0.964	0.129	5.640

Note: US stands for ultrasonic extraction, MS stands for magnetic stirring extraction and NE stands for no enzyme.

**Table 2:** The content of individual phenolic substances under different extraction conditions.

Category	Compound	Retention Time (min)	40 °C and NE (μg/g)	40 °C and 0.5% (μg/g)	40 °C and 1% (μg/g)	50 °C and NE (μg/g)	50 °C and 0.5% (μg/g)	50 °C and 1% (μg/g)
Phenolic acids	Homovanillic acid	26.76	100.42 ± 0.26 ^cd^	139.05 ± 4.43 ^b^	91.74 ±0.88 ^e^	201.14 ± 5.34 ^a^	106.30 ± 0.49 ^c^	213.36 ± 0.26 ^a^
	p-Coumaric acid	34.70	16.22 ± 1.87 ^c^	27.75 ± 1.04 ^c^	15.31 ± 0.27 ^c^	104.67 ± 13.35 ^a^	33.41 ± 9.17 ^c^	60.00 ± 0.33 ^b^
	Chlorogenic acid	23.01	142.92 ± 0.96 ^c^	108.15 ± 9.85 ^e^	99.36 ± 3.13 ^e^	158.11 ± 3.73 ^b^	125.42 ± 7.44 ^d^	240.74 ± 1.20 ^a^
	p-Hydroxybenzoic acid	24.81	23.74 ± 3.05 ^c^	50.50 ± 2.37 ^b^	24.13 ± 3.12 ^c^	47.90 ± 7.90 ^b^	53.34 ± 3.07 ^b^	140.97 ± 5.16 ^a^
	Syringic acid	28.05	34.90 ± 1.42 ^e^	56.84 ± 0.89 ^c^	32.39 ± 0.83 ^e^	148.27 ± 3.74 ^a^	47.00 ± 3.30 ^d^	101.14 ± 2.44 ^b^
	Ferulic acid	35.72	13.75 ± 2.08 ^c^	28.10 ± 1.34 ^b^	12.88 ± 0.65 ^c^	50.62 ± 2.36 ^a^	19.43 ± 3.02 ^bc^	40.57 ± 8.57 ^a^
	Gallic acid	11.84	27.86 ± 0.24 ^b^	12.21 ± 0.07 ^c^	28.23 ± 4.01 ^b^	61.96 ± 0.91 ^a^	38.00 ± 4.52 ^b^	52.86 ± 7.81 ^a^
	Protocatechuic acid	17.95	148.61 ± 3.36 ^b^	174.34 ± 6.91 ^a^	34.64 ± 1.54 ^e^	82.64 ± 2.15 ^c^	43.37 ± 3.22 ^e^	70.91 ± 3.39 ^d^
	Caffeic acid	27.37	78.96 ± 2.40 ^c^	31.49 ± 1.38 ^d^	19.48 ± 0.79 ^e^	171.52 ± 1.82 ^a^	74.47 ± 9.47 ^c^	98.12 ± 3.07 ^b^
Dihydrochalcones	Phloretic acid	30.95	154.32 ± 1.94 ^e^	263.92 ± 6.46 ^c^	161.05 ± 3.66 ^e^	404.97 ± 3.78 ^b^	194.31 ± 13.44 ^d^	438.13 ± 1.34 ^a^
	Phloretin	50.68	-	-	21.49 ± 1.51 ^c^	30.26 ± 5.35 ^b^	8.50 ± 2.02 ^d^	64.34 ± 1.79 ^a^
Flavanols	Procyanidin B2	20.23	136.16 ± 1.86 ^c^	183.73 ± 8.42 ^b^	131.19 ± 3.34 ^c^	328.96 ± 23.37 ^a^	227.33 ± 27.33 ^b^	311.20 ± 18.50 ^a^
	Catechin	20.03	309.10 ± 5.72 ^d^	578.69 ± 14.18 ^b^	539.73 ± 2.50 ^b^	44.08 ± 3.45 ^e^	475.80 ± 45.72 ^c^	855.66 ± 10.93 ^a^
	Epicatechin	25.89	179.66 ± 1.27 ^d^	202.32 ± 33.64 ^d^	169.94 ± 1.44 ^d^	500.73 ± 4.35 ^a^	255.04 ± 12.89 ^c^	413.93 ± 8.69 ^b^
Flavonols	Rutin	40.27	-	-	-	16.88 ± 1.34 ^a^	19.05 ± 3.05 ^a^	20.05 ± 3.88 ^a^
	Quercetin-3-O-rutinoside	43.89	-	-	-	1.46 ± 0.96 ^ab^	2.02 ± 0.97 ^a^	3.06 ± 1.06 ^a^
	Myricetin	43.55	11.19 ± 1.34 ^b^	10.53 ± 0.13 ^b^	9.67 ± 0.57 ^b^	23.74 ± 4.23 ^a^	27.44 ± 3.26 ^a^	26.63 ± 6.63 ^a^

Note: Data were averages of different samples ± standard deviation. Values followed by different letters in each row indicated significant differences (*p* < 0.05).

## Data Availability

The data presented in this study are available on request from the corresponding authors. The data are not publicly available due to privacy restrictions.

## Abbreviations

The following abbreviations are used in this manuscript:

TBH	Tartary buckwheat hulls
NE	No Enzyme
ANFIS	adaptive neuro-fuzzy inference system
ANN	artificial neural network
RSM	surface methodology model
FIS	fuzzy inference system
RMSE	root mean square error (mg/g)
R^2^	coefficient of determination
AAD	absolute average deviation (%)
MFs	membership functions
$D_{e}$	effective diffusion coefficient (m^2^/s)
$C_{s}$	concentration of the TBH phenolic (g/cm^3^)
t	extraction time (min)
x	spherical particle radial coordinate (m)
$C_{L}$	total phenolic content in liquid phase (g/mL)
S	contacting area among the extracting solvent and particles (m^2^)
$V_{L}$	the volume of suspension (mL)
r	particle radius (m)
$C_{s,p}$	total phenolic content from TBH granules predicted by diffusion model (g/mL)
$C_{s,e}$	total phenolic content from TBH granules acquired through experiments (g/mL)
$C_{i,e}$	experimentally determined mass of phenolic extracted from TBH granules (mg/g)
$C_{i,p}$	predicted mass of phenolic extracted from TBH granules (mg/g)
$C_{i,avg}$	the average value of the mass of phenolic extracted from TBH granules across all the experimental data (mg/g)

## Appendix A

Code to simulate the mass transfer process under extraction (an example about extraction of Tartary buckwheat hulls polyphenols under a temperature of 40 °C and an enzyme concentration of 1%)

function xhbwb1

m = 2;

x = linspace (0, 0.00245, 20);

t = linspace (0, 50, 41);

sol = pdepe (m, @pdex1pde, @pdex1ic, @pdex1bc, x, t);

u = sol (:, :, 1);

disp (u);

a = u

b_0 min = mean (a (1, :))

b_150 s = mean (a (3, :))

b_5 min = mean (a (5, :))

b_10 min = mean (a (9, :))

b_15 min = mean (a (13, :))

b_20 min = mean (a (17, :))

b_30 min = mean (a (25, :))

b_40 min = mean (a (33, :))

b_50 min = mean (a (41, :))

surf (x, t, u)

title (‘Numerical solution computed with 20 mesh points.’)

xlabel (‘Distance x’)

ylabel (‘Time t’)

figure

plot (x, u (end, :))

title (‘Solution at t = 2’)

xlabel (‘Distance x’)

ylabel (‘u (x, 2)’)

% --------------------------------------------------------------

function [c, f, s] = pdex1pde (x, t, u, DuDx)

c = 1/ (2.00 ×10^^^(−6));

f = DuDx;

s = 0;

% --------------------------------------------------------------

function u0 = pdex1ic(x)

u0 = 0.0039

% --------------------------------------------------------------

function [pl,ql,pr,qr] = pdex1bc(xl,ul,xr,ur,t)

pl = 0;

ql = 1;

pr = 60 × 0.0002 × 1.1919/ (1.1919 + t) ^^^2;

qr = 2.00 × 10^^^ (−6) × 14,693.87755
